# Supplementary material for: A self-supervised workflow for particle picking in cryo-EM
Source: IUCrJ. 2020 Jun 23;7(Pt 4):719–27. doi: 10.1107/S2052252520007241 (PMC7340252; doi:10.1107/S2052252520007241)
Supplement: Supplementary file 1 [file m-07-00719-sup1.pdf]

IUCrJ

Volume 7 (2020)

Supporting information for article:

A self-supervised workflow for particle picking in cryo-EM

Donal M. McSweeney, Sean M. McSweeney and Qun Liu

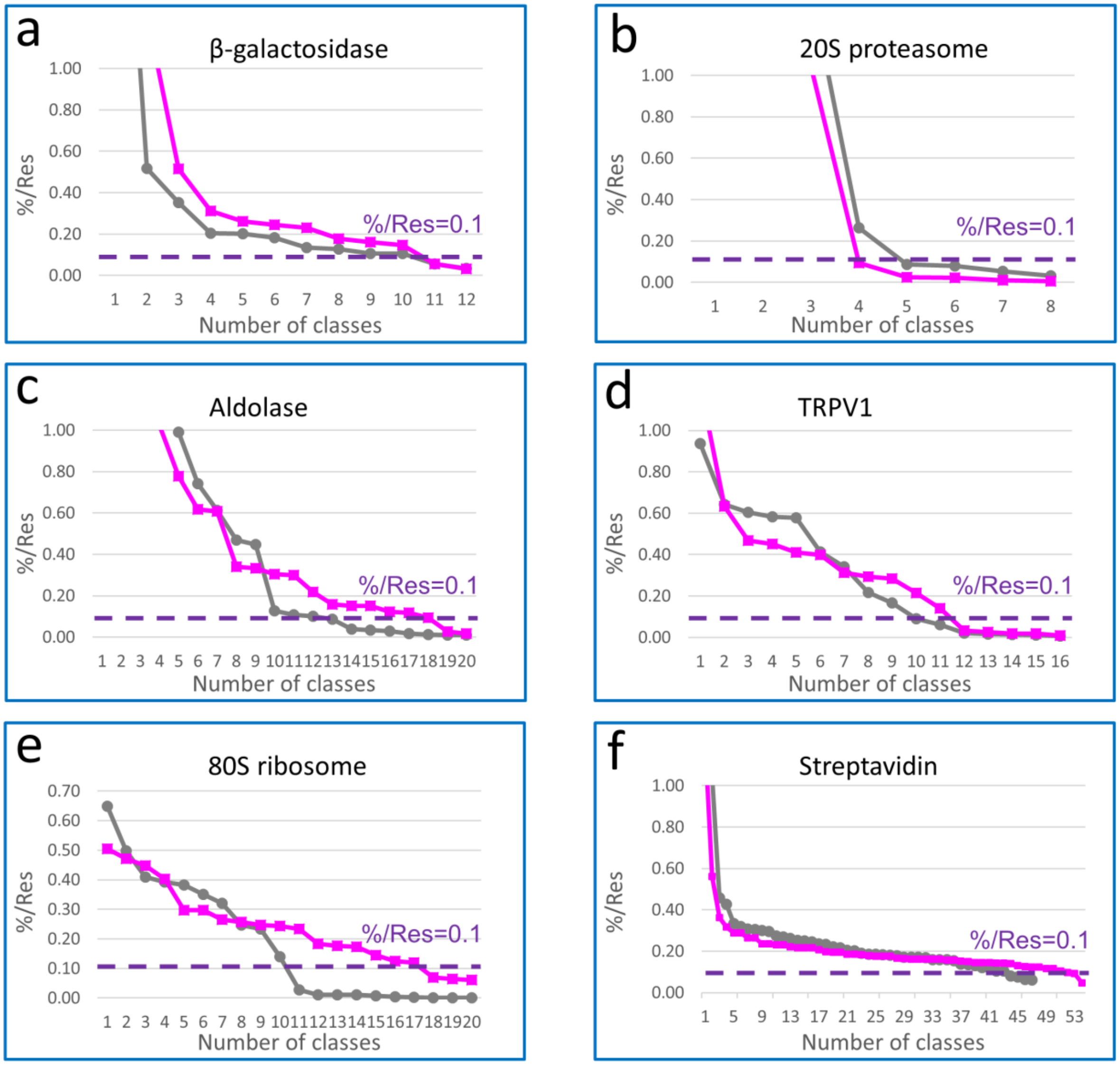

Figure S1. **Comparison of %/Res before and after iterative training and picking in Kpicker for the six EMPIAR data sets**. For each of the six data sets, the distribution of %/Res with respect to the number of 2D classes from particles picked by Localpicker or manually (gray) is compared with that from particles picked by Kpicker (magenta). Dashed purple lines indicate the 0.1 %/Res cutoff that we used for the six test data sets. To highlight the changes of %/Res close to 0.1, values higher than 1.0 were not shown. In general, iterative training and picking yield more classes to be selected, corresponding to more views. Among these test data sets, 20S proteasome is an outline perhaps due to the more preferable side and end views. (a) β-galactosidase, (b) 20S proteasome, (c) aldolase, (d) TRPV1, (e) 80S ribosome, (f) streptavidin.
